# Expressions of Matrix Metalloproteinases (MMP-2, MMP-7, and MMP-9) and Their Inhibitors (TIMP-1, TIMP-2) in Inflammatory Bowel Diseases 

**DOI:** 10.1155/2016/2456179

**Published:** 2016-02-29

**Authors:** Katarzyna Jakubowska, Anna Pryczynicz, Piotr Iwanowicz, Andrzej Niewiński, Elżbieta Maciorkowska, Jerzy Hapanowicz, Dorota Jagodzińska, Andrzej Kemona, Katarzyna Guzińska-Ustymowicz

**Affiliations:** ^1^Department of General Pathomorphology, Medical University of Bialystok, Waszyngtona Street 13, 15-269 Bialystok, Poland; ^2^Department of Rehabilitation, Medical University of Bialystok, Bialystok, Poland; ^3^Department of Developmental Age Medicine and Paediatric Nursing, Medical University of Bialystok, Bialystok, Poland; ^4^Department of Oncological Surgery, Comprehensive Cancer Centre, Bialystok, Poland

## Abstract

Crohn's disease (CD) and ulcerative colitis (UC) belong to a group of inflammatory bowel diseases (IBD). The aim of our study was to evaluate the expression of MMP-2, MMP-7, MMP-9, TIMP-1, and TIMP-2 in ulcerative colitis and Crohn's disease. The study group comprised 34 patients with UC and 10 patients with CD. Evaluation of MMP-2, MMP-7, MMP-9, TIMP-1, and TIMP-2 expression in tissue samples was performed using immunohistochemistry. The overexpression of MMP-9 and TIMP-1 was dominant in both the glandular epithelium and inflammatory infiltration in UC patients. In contrast, in CD subjects the positive expression of MMP-2 and TIMP-1 was in glandular tubes while mainly MMP-7 and TIMP-2 expression was in inflammatory infiltration. Metalloproteinases' expression was associated with the presence of erosions, architectural tissue changes, and inflammatory infiltration in the lamina propria of UC patients. The expression of metalloproteinase inhibitors correlated with the presence of eosinophils and neutrophils in UC and granulomas in CD patients. Our studies indicate that the overexpression of metalloproteinases and weaker expression of their inhibitors may determine the development of IBD. It appears that MMP-2, MMP-7, and MMP-9 may be a potential therapeutic target and the use of their inhibitors may significantly reduce UC progression.

## 1. Introduction

Crohn's disease (CD) and ulcerative colitis (UC) belong to a group of inflammatory bowel diseases (IBD). These are chronic diseases of, as yet, unknown etiology, in which various inflammatory mediators, such as proteolytic enzymes including metalloproteinase, cytokines, and growth factors, and a number of cells including leukocytes and stromal cells are involved [1]. In UC the chronic inflammation, mainly limited to the mucosa of the colon and rectum, may cause crypts and mucosal ulceration [2]. CD may affect the entire gastrointestinal tract, in particular its ileocecal region [3]. Inflammation involves the entire intestinal wall leading to fibrosis and fistulae [2]. In both diseases there exists a significant risk of developing cancer [2].

Metalloproteinases (MMPs) belong to a large group of proteolytic zinc-dependent enzymes which are involved in the remodeling and degradation of extracellular matrix (ECM) by cleavage of one or more of its components. They are synthesized and secreted by cells in an inactive form. The structures of the enzymes are very similar: they consist of a predomain comprising a signal peptide, a catalytic domain containing the zinc binding motif, and a hemopexin-like domain [4]. This family of proteinases presently includes 24 enzymes which have been divided into 6 subgroups based on domain organization and substrate preference: collagenases (MMP-1, MMP-8, and MMP-13), gelatinases (MMP-2, MMP-9), stromelysins (MMP-3, MMP-10, MMP-11, and MMP-18), matrilysins (MMP-7, MMP-26), membranous MMP (MMP-14, MMP-15, MMP-16, MMP-17, MMP-24, and MMP-25), and other MMPs not yet assigned to any group (MMP-11, MMP-12, MMP-19, MMP-20, MMP-23, and MMP-28) [5]. The enzymes are involved both in physiological states such as embryogenesis or wound healing and in pathological conditions, for example, rheumatoid arthritis, atherosclerosis, and tumor metastasis [6–8].

The activity of the metalloproteinases is controlled by macroglobulins and, predominantly, Tissue Inhibitors of Metalloproteinases (TIMPs). There are currently four known inhibitors referred to as TIMP-1, TIMP-2, TIMP-3, and TIMP-4 [9]. They consist of two structurally and functionally distinct domains. The N-terminal domain is an effective inhibitor of all MMPs through binding to the enzymes' catalytic portion containing zinc. The C-terminal domain has at least two separate enzyme binding sites, one for gelatinases and the other one for stromelysins [9]. Tissue inhibitors affect all MMPs with the exception of TIMP-1 which does not inhibit MT1-MMP (Membrane-Type 1-Matrix Metalloproteinase). Properties of TIMP-3, however, are distinguished from the remaining tissue inhibitors as studies have proven it to be a better inhibitor of ADAM-17 (TACE), ADAM-10, and ADAM-12 than of MMP [10]. The inhibitors bind to MMPs in a stoichiometric ratio of 1 : 1. The expression of TIMPs as well as MMPs must be controlled to maintain physiological functions in tissue remodeling. Disturbances of this balance may lead to diseases of uncontrolled ECM component formation such as neurological and cardiovascular diseases, ulcers, and tissue fibrosis [9]. Still there is little known about expression of MMP-2, MMP-7, MMP-9, TIMP-1, and TIMP-2 in IBD patients, which may help to better understand the disease and to be used as therapeutic targets.

The aim of our study was to evaluate the immunohistochemical expression of MMP-2, MMP-7, MMP-9, TIMP-1, and TIMP-2 in patients with UC and CD in correlation with histo- and clinicopathological parameters.

## 2. Materials and Methods

### 2.1. Materials

The study was performed in conformity with the Declaration of Helsinki for Human Experimentation and received approval by the Local Bioethics Committee of the Medical University of Bialystok.

The study groups consisted of 34 patients (25 male, 9 female) with UC and 10 patients (7 male, 3 female) with CD. The study materials were obtained from core needle biopsy, embedded in paraffin blocks acquired in the Second Department of General Surgery and Gastroenterology at the Medical University of Bialystok in the years 2003–2005. Study included 17 patients under the age of 18 diagnosed with UC and none with CD. We found 17 patients with UC and 10 with CD to be over 18 years of age.

### 2.2. Histopathological Examination

Sections were stained with hematoxylin and eosin (H&E) and subjected to routine histopathological assessment. The presence of epithelial dysplasia was noted and classified as negative, indefinite, low, and high grade. Indefinite dysplasia was observed in 15 cases of UC and in 5 cases of CD, whereas low-grade dysplasia was observed in 10 cases and high-grade dysplasia in 3 cases of UC. Low-grade dysplasia was present in only 1 case of CD. Disease activity was assessed according to the Geboes criteria [11]. Inactive disease was observed in 9 cases of UC and in 2 cases of CD. Active inflammation was present in 5 cases of UC and 4 cases of CD, whereas chronic disease was noted in 20 cases of UC and 4 cases of CD (Table 1).

### 2.3. Immunohistochemistry

Formalin-fixed, paraffin-embedded tissue slides were cut on a sliding microtome into 4 *μ*m thick sections. Sections were deparaffinized in serial xylene and rehydrated in alcohol. Only the MMP-9 antigen was not diluted in any buffer. To visualize the antigens of MMP-2 and TIMP-1, sections were heated in a microwave oven for 20 min in EDTA buffer at pH = 9. TIMP-2 was treated with citrate buffer (pH 6). Endogenous peroxidase was blocked for 5 minutes. Next, they were incubated with anti-human antibodies: mouse monoclonal antibody of Matrix Metalloproteinase 2 (clone 17B11, Novocastra, UK; dilution 1 : 60); mouse monoclonal antibody of Matrix Metalloproteinase 7 (clone 111433, R&D Systems, USA; dilution 1 : 75); mouse monoclonal antibody of Matrix Metalloproteinase 9 (clone 15W2, Novocastra, UK; dilution 1 : 80); mouse monoclonal antibody of Tissue Inhibitor of Matrix Metalloproteinase 1 (clone 6F6a, Novocastra, UK; dilution 1 : 150); mouse monoclonal antibody of Tissue Inhibitor of Matrix Metalloproteinase 2 (clone 46E5, Novocastra, UK; dilution 1 : 20). In each case, incubation continued for 1 hour at room temperature. Antibodies for metalloproteinase and their inhibitors were specific for human pro and active forms. Following the reaction in the streptavidin-biotin system (Biotinylated Secondary Antibody, Streptavidin-HRP, Novocastra, UK) the antigen-antibody complex was visualized with the use of chromogen 3,3-diaminobenzidine (DAB, Novocastra, UK). All sections were counterstained with hematoxylin.

### 2.4. Statistical Analysis

The statistical analysis was conducted using the STATISTICA 10.0 program (StatSoft, Cracow, Poland). Student's *t*-test was used to compare the two groups. Correlations between the parameters were calculated with Spearman's rank correlation coefficient tests. The *p* value < 0.05 was considered statistically significant.

## 3. Results

### 3.1. Expression of Matrix Metalloproteinases (MMP-2, MMP-7, and MMP-9) in Glandular Epithelium and Inflammatory Cells of UC and CD

**Figure 1:**
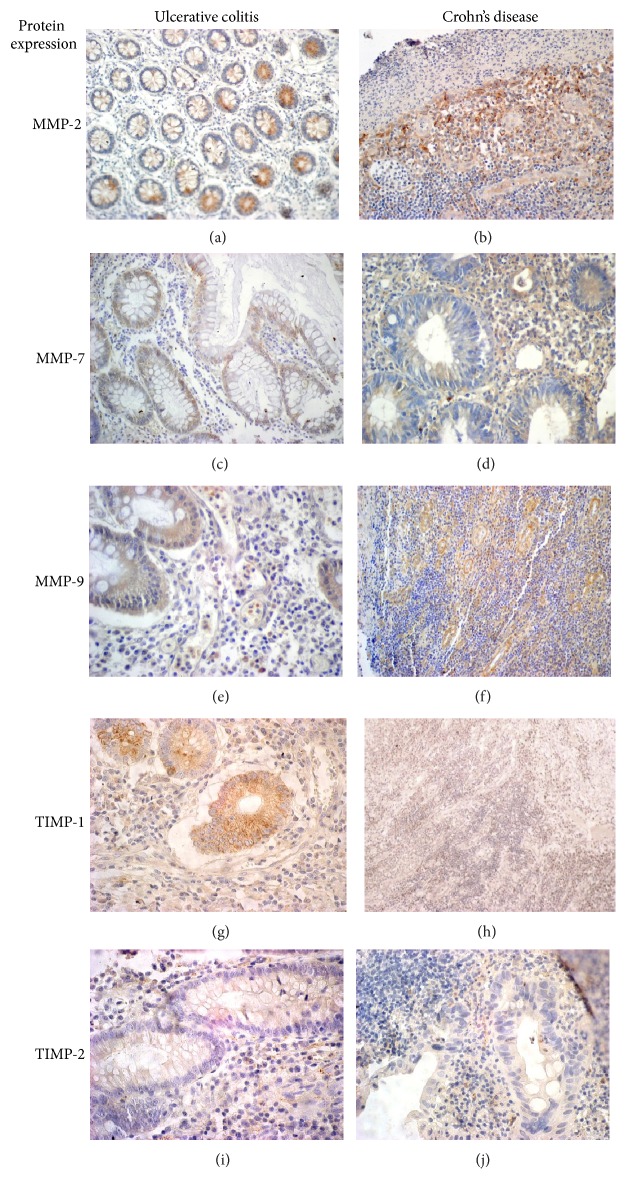
Immunohistochemical expression of MMP-2, MMP-7, MMP-9, TIMP-1, and TIMP-2 in glandular tubes and inflammatory cells in tissues of ulcerative colitis (*N* = 34) and Crohn's disease (*N* = 10). MMP-2 expression was weak in glandular tubes in patients with UC and positive reaction of this protein in inflammatory cells of CD (a, b). The positive expression of MMP-7 in glandular cells in both diseases, but there is more frequent expression observed in stroma of CD (c, d). Moreover, MMP-9 reaction was strong positive in glandular epithelium of UC and moderate in the stromal cells of CD patients (e, f). The strong expression of TIMP-1 in glandular cells and inflammatory infiltrate in both diseases compared to lack of or weak reaction of TIMP-2 protein (g, h, i, and j).

A weak expression of MMP-2 was dominated in both the inflammatory infiltration and glandular epithelium of 73.3% of patients with UC. Moderate and high reactions of MMP-2 protein were present in a greater proportion in glandular tubes (16.7% and 6.7%, resp.) than in the inflammatory cells (9.1% and 0%, resp.). In Crohn's disease, the expression of MMP-2 in the glandular epithelium was strong in 60% of cases while its reaction in inflammatory cells was weak in 81.8% of cases. The expression of MMP-2 in glandular epithelium was significantly higher in CD compared to UC (*p* = 0.009). The expression of MMP-7 in the glandular epithelium was absent in 54.9% as opposed to the expression observed in inflammatory cells (weak: 35.5%, moderate: 32.3%, and strong: 25.8%). We found a weak expression in the glandular epithelium (60%) and strong reaction in inflammatory infiltration in patients with CD (60%). The expression of MMP-9 in patients with UC was weak in 33.3% of glandular epithelium and in 32.3% of inflammatory infiltration. Strong expression of MMP-9 was observed in 38.7% with inflammatory infiltration and 36.7% in the glandular epithelium. We found weak expression of MMP-9 in glandular epithelium (50% of cases) and in 41.7% in inflammatory infiltration of CD patients (Figure 1). Expression of MMP-9 was statistically higher in both glandular epithelium and inflammatory infiltration of UC compared to CD patients (*p* = 0.042, *p* = 0.003). (Results are shown in Table 2.)

### 3.2. Expression of Matrix Metalloproteinase Inhibitors (TIMP-1, TIMP-2) in Glandular Epithelium and Inflammatory Cells of UC and CD

We found strong expression of TIMP-1 in 62.5% of cases of glandular epithelium and in 37.5% of cases in cells of inflammatory infiltration in patients with UC. Expression of TIMP-1 was strong in 66.7% of the glandular epithelium in contrast with weak reaction in 55.6% of patients with CD.

A positive reaction of TIMP-2 was higher in the glandular epithelium than in infiltration of inflammatory cells in patients with UC. We found weak expression of TIMP-2 in the glandular epithelium (62.5%) and the absence or favorable reaction of TIMP-2 in infiltration of inflammatory cells (66.7% of cases) (Table 3).

### 3.3. The Correlation between Expression of Matrix Metalloproteinases (MMP-2, MMP-7, and MMP-9) and Histopathological Parameters

The statistical analysis showed a positive correlation between MMP-2 expression in the glandular epithelium of UC patients and the presence of erosions or ulcers (*p* = 0.048, *R* = 0.377). There was also a trend of increased expression of MMP-2 in the glandular epithelium concurrent with progression of changes in tissue architecture and the presence of neutrophils in the lamina propria (*p* = 0.073, *R* = 0.344; *p* = 0.074, *R* = 0.349, resp.). There was a correlation between the predominant weak expression of MMP-2 in the inflammatory infiltration and the presence of neutrophils in the lamina propria (*p* = 0.041, *R* = 0.388). No correlation was found between MMP-2 expression in the glandular epithelium and inflammatory infiltration in CD patients and the histopathological features.

The expression of MMP-7 in the glandular epithelium of UC patients positively correlated with the occurrence of erosions (*p* = 0.027, *R* = 0.449). In CD patients, a strong positive correlation was found between MMP-7 expression in the glandular epithelium and the location of the lesion (*p* = 0.000, *R* = 0.898).

No statistically significant relationship was established between MMP-9 in the glandular epithelium and inflammatory infiltration in patients with UC and CD. We observed a trend towards decrease in expression of MMP-9 protein and marked architectural tissue changes in patients with UC (*p* = 0.064, *R* = −0.361).

### 3.4. The Correlation between Expression of Matrix Metalloproteinase Inhibitors (TIMP-1, TIMP-2) and Histopathological Parameters

In UC patients, MMP-2 expression in the lamina propria of inflammatory infiltration correlated with presence of neutrophils, whereas TIMP-1 expression depended on the presence of eosinophils in the lamina propria (*p* = 0.021, *R* = 0.588) and neutrophils in the glandular epithelium (*p* = 0.029, *R* = 0.0563). In contrast, in CD patients TIMP-1 expression in the glandular epithelium was inversely related to the patients' age (*p* = 0.049, *R* = −0.669) while its response in the inflammatory infiltrate was associated with the presence of granulomas (*p* = 0.016, *R* = 0.848).

The statistical analysis of TIMP-2 expression in the glandular epithelium of UC patients showed a significant correlation with the age of patients (*p* = 0.029, *R* = 0.466) who were divided into two groups: 1: <18 and 2: ≥18 years of age. In group 2, expression was moderate whereas in group 1 weak expression was dominant. We did not observe a relationship between TIMP-2 in the glandular epithelium and inflammatory cells and histopathological features.

## 4. Discussion

A series of destructive as well as regenerative processes occur in both UC and CD. Different levels of metalloproteinase and their inhibitor expression are observed depending on their stage of advancement. Our studies have shown a tendency towards weak expression of MMP-7 in glandular epithelium and a predominantly moderate or strong response in inflammatory infiltration in approximately 60% of UC patients. In contrast, Rath et al. [12] and Newell et al. [13] demonstrated an increase in MMP-7 mRNA levels which correlated with disease severity and degree of dysplasia in UC patients. Our study has shown positive expression of MMP-7, both in the glandular tubes and in inflammatory infiltrate in all CD patients which increased with the incidence of erosions. Inflammatory cells including neutrophils which are responsible for maintaining a local inflammatory response and stromal degradation may be the source of MMP-7.

Our research has demonstrated mainly weak expression of MMP-2 in the inflammatory infiltration and a much stronger response in the glandular epithelium of patients with UC and CD. von Lampe et al. [14] and Pirilä et al. [15] also confirmed the overexpression of MMP-2 in colonic mucosa of UC patients. Furthermore, Sim et al. [16] observed the upregulation of MMP-2 mRNA in patients with CD. It has been proven that the overexpression of MMP-2 in cultured intestinal epithelial cells determines the integrity of the protective barrier whereas its downregulation affects the sensitivity of the mucosa and may determine the occurrence of colitis [17]. In our study, we have observed that the expression of MMP-2 in glandular epithelium correlated with the presence of erosions. By contrast, MMP-2 immunoreactivity in the inflammatory infiltrate in UC patients positively correlated with the presence of neutrophils in the lamina propria. MMP-2 expression in both glandular epithelium and inflammatory infiltration, dependent mainly on mesenchymal cells, neutrophils, and eosinophils, determines the disorganization of protective structures, the decomposition of collagen types IV and V, and the degradation of the stromal tissue of IBD patients [18, 19].

Continuous inflammatory response in the intestinal mucosa in the course of IBD appears to be an important therapeutic target. The majority of animal colitis models have been based on chemically induced models with the dextran sulfate sodium-induced colitis model being the most widely used due to its similarities with human ulcerative colitis. In experimental studies, the dextran sulfate sodium- (DSS-) induced colitis has shown a lack of MMP-9 expression in healthy intestinal mucosa which is upregulated in inflamed mucosa of IBD [20]. DSS-induced colitis studies have proved that MMP-9 activity increases in homogenates of colonic mucosa in UC and is dependent on TNF-alpha [21, 22]. Studies in animal models have confirmed that the lack of MMP-9 −/− expression in the DSS-induced colitis determines a reduction in inflammation and damage to the intestinal mucosa [23, 24]. In our study, we have observed mainly positive expression of MMP-9 in both glandular epithelium and inflammatory infiltration in UC. Mao et al. [21] also demonstrated significantly higher expression of MMP-9 in the colonic mucosa of UC patients compared to the control group. In addition, activity of the protein has increased in homogenates of the inflamed mucosa of both UC and CD [25]. Furthermore, the statistical analysis of our research data has confirmed a tendency towards increased MMP-9 expression in UC patients who displayed changes in the architecture of the colonic tissue. It has been proved that MMP-9 is produced by a variety of inflammatory cells, in particular polymorphonuclear leukocytes (PMNL), and secreted in response to local inflammation [26]. We therefore believe that the lasting activation of the MMP-9 protein expression and the chronic inflammation of the lining of the colon in UC patients may lead to a loss of structural tissue integrity and may determine tissue damage. In contrast to UC patients, we have demonstrated low expression of MMP-9, in particular in the inflammatory infiltration, and its slightly higher immunoreactivity in the glandular tubes in CD patients. Our observations are contrary to Bailey et al. [20] who observed positive reaction of MMP-9 in PMNL present in the lamina propria more and less in the submucosa and muscularis propria. Our findings and the available literature reports suggest that MMP-9 plays an important role in the pathogenesis of colitis and may be a potential target for anti-inflammatory therapy [27, 28].

TIMP-1 and TIMP-2 are responsible for controlling the activity of Matrix Metalloproteinases, thus maintaining the correct balance in the remodeling and degradation of ECM. Wang and Yan [29] reported positive expression of TIMP-1 in 80–89% of cases of inflamed ulcerative changes and intact colon mucosa in UC patients and in 75% of cases of normal colon mucosa. Rath et al. [12] reported significantly higher presence of TIMP-1 in the inflamed mucosa of adult IBD patients. In contrast, Mäkitalo et al. [30, 31] confirmed positive expression of TIMP-2 in epithelial cells and stroma in adult patients while lack of expression of TIMP-1 was found in all the studied cases of pediatric UC patients. Despite their enhanced activity in both diseases, it appears that tissue inhibitors expressions are too weak or their activation occurs too late to prevent the development of either condition.

The immunohistochemical analysis of our research data indicates that the overexpression of metalloproteinases and much weaker activation of the inhibitors in tissue samples may determine the development of IBD. A significant correlation has been established in UC patients, in particular between the increased expression of metalloproteinases and the examined histopathological markers which determine disease progression. It seems that MMP-7, MMP-2, and MMP-9 may be potential therapeutic targets and the use of their inhibitors may significantly reduce disease progression in UC patients. Nevertheless, the present findings should be confirmed in a larger study group in the future.

## Figures and Tables

**Table 1:** Demographic and histological characteristics of the study group.

Disease	*N*	Age		Gender		Localization			Grade of dysplasia				Disease activity		
		<18	>18	F	M	1	2	3	N	IN	L	H	IA	A	CH
Ulcerative colitis (UC)	34	17	17	9	25	14	7	13	8	15	10	3	9	5	20
Crohn's disease (CD)	10	0	10	3	7	6	3	1	4	5	1	0	2	4	4

F: female, M: male; 1: proctitis in UC, colon in CD, 2: left-sided colitis in UC, colon + rectum in CD, and 3: pancolitis in UC, rectum in CD; grade of dysplasia: N: negative, IN: indefinite, L: low, and H: high; disease activity: IA: inactive, A: active, and CH: u

**Table 2:** Immunohistochemical expression of MMP-2, MMP-7, and MMP-9 in glandular tubes and inflammatory cells in tissues of ulcerative colitis and Crohn's disease.

Protein expression	Ulcerative colitis (UC)								Crohn's disease (CD)							
	*Glandular cells* (*% of cases*)				*Inflammatory cells* (*% of cases*)				*Glandular cells* (*% of cases*)				*Inflammatory cells* (*% of cases*)			
	0	1	2	3	0	1	2	3	0	1	2	3	0	1	2	3
MMP-2	3.3	73.3	16.7	6.7^1^	18.2	72.2	9.1	0	20	10	10	60^2^	9.1	81.8	9.1	0
MMP-7	54.9	29	16.1	0	6.4	35.5	32.3	25.8	0	60	30	10	0	20	20	60
MMP-9	6.7	33.3	23.3	36.7^3^	3.2	32.3	25.8	38.7^5^	16.7	50	25	8.3^4^	33.3	41.7	16.7	8.3^6^

1  versus 2 = 0.009; 3 versus 4 = 0.042; 5 versus 6 = 0.003.

0: absent, 1: weak, 2: moderate, and 3: strong.

**Table 3:** Immunohistochemical expression of TIMP-1 and TIMP-2 in glandular tubes and inflammatory cells in tissues of ulcerative colitis and Crohn's disease.

Protein expression	Ulcerative colitis (UC)								Crohn's disease (CD)							
	*Glandular cells* (*% of cases*)				*Inflammatory cells* (*% of cases*)				*Glandular cells* (*% of cases*)				*Inflammatory cells* (*% of cases*)			
	0	1	2	3	0	1	2	3	0	1	2	3	0	1	2	3
TIMP-1	18.8	12.5	6.3	62.5	25	31.2	6.3	37.5	22.2	11.1	0	66.7	22.2	55.6	22.2	0
TIMP-2	10	45.5	31.8	18.2	40.9	31.8	9.1	18.2	12.5	62.5	12.5	12.5	66.7	11.1	0	22.2

0: absent, 1: weak, 2: moderate, and 3: strong.
